# Community-based HIV testing through a general health check event in a high HIV-prevalent multicultural area in Rotterdam, The Netherlands: a pilot study on feasibility and acceptance

**DOI:** 10.1186/s40814-023-01327-w

**Published:** 2023-06-16

**Authors:** Denise E. Twisk, Anita Watzeels, Hannelore M. Götz

**Affiliations:** 1 Department of Public Health, Municipal Public Health Service Rotterdam-Rijnmond, Rotterdam, The Netherlands; 2 Department of Public Health, Erasmus MC, University Medical Center Rotterdam, Rotterdam, The Netherlands; 3 Department Research and Business Intelligence, Municipality of Rotterdam, Rotterdam, The Netherlands; 4 Centre for Infectious Disease Control, National Institute for Public Health and the Environment (RIVM), Bilthoven, The Netherlands

## Abstract

**Background:**

HIV testing is crucial for finding the remaining cases in a declining HIV epidemic in The Netherlands; providing HIV testing in non-traditional settings may be warranted. We conducted a pilot study to determine the feasibility and acceptability of a community-based HIV testing (CBHT) approach with general health checks to improve HIV test uptake.

**Methods:**

CBHT’s main conditions were low-threshold, free-of-charge, general health check, and HIV education. We interviewed 6 community leaders, 25 residents, and 12 professionals/volunteers from local organizations to outline these main conditions. Walk-in test events were piloted at community organizations, providing HIV testing along with body mass index (BMI), blood pressure, blood glucose screening, and HIV education (October 2019 to February 2020). Demographics, HIV testing history, risk perception, and sexual contact were collected via questionnaires. To evaluate the pilots’ feasibility and acceptance, we utilized the RE-AIM framework and predefined goals, incorporating quantitative data from the test events and qualitative input from participants, organizations, and staff.

**Results:**

A total of 140 individuals participated (74% women, 85% non-Western, median age 49 years old). The number of participants during the seven 4-h test events ranged from 10 to 31. We tested 134 participants for HIV, and one was found positive (positivity 0.75%). Almost 90% of the participants were never tested or > 1 year ago, and 90% perceived no HIV risk. One-third of the participants had one or more abnormal test results on BMI, blood pressure, or blood glucose. The pilot was well-rated and accepted by all parties. The staff had concerns about waiting time, language problems, and privacy. Participants hardly indicated these concerns.

**Conclusions:**

This CBHT approach is feasible, acceptable, and well-suited for testing not (recently) tested individuals and detecting new cases. Besides reducing HIV-associated stigma and increasing HIV test acceptance, offering multiple health tests may be appropriate as we frequently observed multiple health problems. Whether this laborious approach is sustainable in the micro-elimination of HIV and should be deployed on a large scale is questionable. CBHT like ours may be suitable as a supplement to more sustainable and cost-effective methods, e.g., proactive HIV testing by general practitioners and partner notification.

## Key messages regarding feasibility

- **What uncertainties existed regarding the feasibility?**

HIV testing continues to be a key strategy in case finding. Providing HIV testing in non-traditional settings may be warranted in the micro-elimination of HIV in The Netherlands. There are limited studies on community-based HIV testing and multi-disease testing approaches in high-income countries, like The Netherlands.

- **What are the key feasibility findings?**

Offering a rapid HIV test combined with other health tests at community organizations was feasible, acceptable, and well-rated by participants, stakeholders, and staff. The approach was effective in testing not (recently) tested persons and detecting new cases.

- **What are the implications of the feasibility findings for the design of the main study?**

The results of this study inform how to design and conduct community-based HIV testing with a multi-disease approach. However, it is unclear whether this laborious approach outweighs finding the last fraction of unidentified HIV cases, and whether it should be conducted on a large scale.

## Background

Over the last decade, significant efforts have been made to tackle the global HIV epidemic, especially after the introduction of the UNAIDS 90–90-90 targets [1]. The UNAIDS targets have been recently revised to 95–95-95 by 2025 [2]. The Netherlands nearly achieved the 95–95-95 targets in 2019; 93% of people living with HIV (PLHIV) are aware of their HIV status, 93% of those diagnosed are on antiretroviral therapy, and 96% of those under treatment have viral suppression [3]. However, there are large regional differences in the Dutch HIV epidemic, with nearly half of all PLHIV residing in one of the four largest cities of The Netherlands, including Amsterdam and Rotterdam [3].

With the declining HIV epidemic in The Netherlands, we currently enter the phase of micro-elimination of HIV. A geographically targeted approach may be warranted and more effective as HIV prevalence varies greatly across the country, even within regions and cities [4]. While focusing on more regional approaches, HIV testing continues to be a key strategy. Many barriers undermine HIV testing uptake at the individual (e.g., low-risk perception, fear of disease, discrimination and judgment, limited knowledge), healthcare provider (e.g., no proactive testing, unease to discuss HIV/sexual behavior, fear of discriminating, insufficient time), and health service (e.g., location, waiting time, costs) levels [5–7]. To overcome these barriers, other approaches are introduced to increase HIV testing. Outreach community-based HIV testing (CBHT) is seen as an acceptable and effective strategy to overcome most provider and health service level barriers, and thereby reach populations not accessing healthcare settings and/or populations that have not recently or never tested before [8]. However, individual barriers like fear of stigma could still affect CBHT participation. Integration of HIV testing into a broader service delivery with less stigmatized non-communicable diseases (e.g., hypertension screening) could normalize HIV testing and thus reduce stigma and increase HIV test uptake [9, 10].

In contrast to several low- and middle-income countries, CBHT interventions combining HIV testing with other general health tests are infrequently documented for high-income countries [8, 11–13]. To our knowledge, The Netherlands limits outreach activities on HIV testing to occasional events and mostly targets high-risk groups, for example, around World AIDS Day [14, 15]. The Public Health Service (PHS) of Rotterdam aimed to assess the feasibility and acceptability of an intervention to improve HIV test uptake in the general population by offering an HIV test combined with more general health tests in a community setting in the city of Rotterdam, The Netherlands. To evaluate the feasibility and acceptance, we utilized the RE-AIM framework that describes the reach, effectiveness, adaptation, implementation, and maintenance of the pilot [16].

## Methods

### Study design

This pilot study employed an observational cross-sectional design. For the evaluation, we applied the RE-AIM framework with both quantitative and qualitative data.

### Input stakeholders

We conducted a pilot CBHT intervention (hereafter, test event) with a community participatory approach to improve HIV testing uptake. First, we selected an intervention area (neighborhood) in Rotterdam that ranked as highest on HIV prevalence (6.6 per 1000 residents) and proportion of residents with a non-western migratory background (66.0%), an important key population in The Netherlands [3, 17, 18]. Second, individual semi-structured interviews were conducted with community leaders (*N* = 6), residents (*N* = 25), and professionals and volunteers from local organizations (*N* = 12) from the selected area to solicit advice on the design and implementation of the test events. Stakeholders were recruited through snowball sampling (i.e., recruiting within stakeholders’ social networks). Interviews were transcribed verbatim. Barriers and facilitators for HIV testing were identified via inductive qualitative content analysis. The stakeholders’ recommendation to improve HIV testing uptake included the following:

1. Combine HIV testing with more general health tests to overcome HIV-related taboo/stigma
2. Create a low-threshold setting by offering free-of-charge anonymous rapid HIV tests at non-medical locations that residents already visit and link up with existing activities, which ensures relatively little effort for residents and an opportunity to test unseen
3. Include HIV education since knowledge was estimated as low

Based on these suggestions, a general health check test event was designed (HIV test, body mass index (BMI), blood pressure (BP), and blood glucose (BG)). We conducted a trial run before the pilot launch. This pre-testing took place in the week before World AIDS Day 2018. No major adjustments were necessary.

### Procedures

Seven 4-h walk-in test events were held at three community organizations (October 2019 to February 2020): once at a boxing school, three times at a community center, and three times at a community support organization where mainly women with a migratory background come to socialize.

Test events were announced by community leaders (e.g., word of mouth, social media, and community organizations’ website), via the PHS website and social media, and by posters and leaflets in the neighborhood among others at locations of interviewed community organizations. If walk-in was considered as low, passers-by and people present at nearby organizations were actively invited.

Each eligible person (≥ 18 years) that walked in was informed about the test event and procedure. Those who declined to participate were asked for their reason (non-participants). Participants filled out an informed consent form. The test event was organized in three different stops. First, a questionnaire was administered orally and anonymously by a researcher with questions about sociodemographics, HIV testing behavior, HIV risk perception, and sexual contact. In addition, we asked women if they had children and the children’s years of birth. Women with pregnancy after 2003 have most likely been tested for HIV as part of a national HIV screening program [19]. At the second stop, BMI was computed with weight and height measures, and BP was checked. The last stop included BG measurement and HIV testing via a finger-prick blood sample. We used the INSTI™ HIV1/HIV2 Rapid Antibody test (Biolytical TM, Laboratories Inc., Richmond, BC, Canada), which yields the HIV test result within 1 min. A sexual health nurse of the PHS performed the HIV test, communicated the HIV test result, and provided counseling. Participants received a record of their test results including links to reliable health websites and were given verbal health advice. If results on BMI, BP, or BG fell outside the recommended guidelines, and participants were not yet aware of this, they received a letter for their general practitioner (GP) [20–22]. A positive rapid HIV test was followed by a consultation at the PHS within 24 h. After rapid laboratory confirmation of the HIV infection, the participant received counseling and was referred to specialized HIV care according to regular procedures. All services were provided free of charge and anonymously. Before the start and in between stops, waiting time could be filled with an educational true or false game with facts and myths about HIV. The game was led by a health educator or a peer living with HIV, who would then discuss the answers with the participants. The peer was also present for counseling after a positive rapid HIV test.

### Evaluation

To guide our evaluation, we utilized the RE-AIM framework. To examine *Reach*, the demographic characteristics of the study population are described and compared to the target population, i.e., the residents within the selected geographical area. The study population is also described in terms of their health and HIV testing history, as this reflects the value of targeting this population, and whether they might benefit from health checks. Predefined quantitative goals were assessed to determine whether the pilot had the ability to reach the preferred population. Table 1 gives an overview of these goals and the rationale behind the goals. The proportion of non-participants and the reason for non-participation are provided as well. The outcome *Effectiveness* is embodied by the uptake of HIV testing by participants at the test events. Indicators of acceptability, and perceived usefulness of the intervention design (e.g., actively offered, part of general health check), are also included. For *Adaption*, the willingness to participate and engagement of community organizations was used as indicators. The focus of *Implementation* is on the key successes and challenges of the test events, based on feedback from participants, community leaders, and staff involved in events. The staff and community leaders’ experiences with the test events are also explored. *Maintenance* is operationalized as the value and willingness to continue the pilot according to community leaders and staff.

**Table 1:** Overview of predefined quantitative goals regarding the preferred population and the goal rationale

No	Goal	Goal rationale
1	Minimum of 25 persons per test event	Number of participants during the pre-test (*N* = 25) and estimated capacity to include in all stops in 4 h
2	No selective reach	Sociodemographics of participants reflects the area’s composition based on sex, age, and migratory background [18]
3	70% first-time HIV testers	• Two national representative studies from The Netherlands and Britain that reported 16–25% were tested at least once for HIV (i.e., 75–84% never tested) [23, 24] • We downscaled the proportion of first-time testers to 70%, because our pilot was conducted in a highly urbanized area that harbors a relatively high proportion of non-western residents, which is associated with higher proportions of people who have been tested at least once [23, 24]
4	80% not recently tested for HIV (i.e., > 12 months)	Two national representative studies from The Netherlands and Britain that reported 14–23% of the population was tested in the last year (i.e., 77–86% not recently tested) [23, 24]
5	HIV positivity of 0.33–0.66%	Comparable to the area’s HIV positivity (0.33–0.66%) [17]

For the RE-AIM dimensions and indicators, we used the following quantitative and qualitative data sources:

1. Quantitative data collected during the test events, such as questionnaires from participants and health results from participants *(Reach, Effectiveness)*
2. A short smiley-rated questionnaire among participants (not good, neutral, good, very good), evaluating different aspects of the program, including location, waiting time, staff, provided information, the combination HIV test with other health tests, general atmosphere, and privacy *(Effectiveness, Implementation)*
3. Interviews with the community leader on location about the experiences of the test event *(Reach, Effectiveness, Adoption, Implementation, Maintenance)*
4. Questionnaires among staff about experiences and whether the events had been performed as intended *(Reach, Effectiveness, Adoption, Implementation, Maintenance)*
5. One closing focus group discussion (FGD) with the key staff. All evaluation questionnaires and interviews served as input for the FGD *(Reach, Effectiveness, Adoption, Implementation, Maintenance)*

### Data analysis

Quantitative data included participant questionnaires (*N* = 140), health results (*N* = 138), and participant smiley ratings (*N* = 115). These data were anonymously registered and analyzed using SPSS (version 26). All data were categorical, and age was condensed into four categories standardly used by Statistics Netherlands. Due to the small number of participants, detailed subgroup analysis could not be performed (e.g., by sex and migratory background). Qualitative data were collected from staff questionnaires (*N* = 29), interviewer field notes from interviews with community leaders (*N* = 7), and one FGD with staff. Key themes were extracted from the free text responses in questionnaires and interviewer field notes through document analysis using an inductive process. The FGD was transcribed verbatim, and themes were identified using content analysis.

### Ethics statement

The Medical Ethics Committee of the Erasmus Medical Center, Rotterdam, The Netherlands, decided that this study did not require Institutional Review Board approval (MEC-2019–0431). All participants signed written informed consent after they were made clear that participation in this study was voluntary and anonymous and that they could refuse or discontinue their participation at any time.

## Results

A total of 178 persons were registered as either participants (*n* = 140; 78.7%) or non-participants (*n* = 38; 21.3%) during seven 4-h walk-in test events at three community organizations between October 2019 and February 2020. Not every participant answered all questions in the questionnaire and did all health tests, due to language barriers or time constraints by the participant.

### Reach

#### Participants’ sociodemographics

The number of participants per event ranged from 10 to 31. Based on the number of participants during the pre-test, we aimed for a minimum of 25 persons per test event. This was only achieved at two of the seven test events (Table 2).

**Table 2:** Achievement of predefined goals

No	Goal	Goal achievement
1	Minimum of 25 persons per test event	Partially achieved: 2 out of the 7 test events had ≥ 25 participants (26 and 31 participants), the remaining test events had 19 participants (3 times), 16 participants, or 10 participants
2	No selective reach	Not achieved (Table 3)
3	70% first-time HIV testers^a^	Not achieved: • Not corrected: 65.4% (*n* = 83/127) • Corrected^b^: 52.8% (*n* = 67/127)
4	80% not recently tested for HIV (i.e., > 12 months)^c^	Achieved: • Not corrected: 80.0% (*n* = 28/35)^d^ • Corrected^b^: 87.3% (*n* = 48/55)^d^
5	HIV positivity of 0.33–0.66%	Achieved: 0.75%; 95% CI: 0.02–4.09% (*n* = 1/134)

*CI* Confidence interval

^a^Among all participants tested for HIV at the test events and that had information on HIV testing history

^b^We asked women if they had children and the children’s years of birth to correct for national HIV screening among pregnant women (pregnancy after 2003). Women who had a child after 2003 but did not report being tested for HIV were reclassified into the tested group

^c^Among all participants previously tested for HIV and that had information on HIV testing history

^d^Five participants were excluded for whom the duration since their last HIV tests was unknown

The sociodemographics of the 140 participants are presented in Table 3. The most common non-Western migratory backgrounds were Moroccan (*n* = 43/119, 36.1%), Sub-Sahara African (*n* = 23/119, 19.3%), Surinamese (*n* = 22/119, 18.5%), and Turkish (*n* = 19/119, 16.0%). The participants were not representative of the residents within the selected geographical area. Participants were more often women, non-Western, and middle-aged (Table 3).

**Table 3:** Sociodemographic characteristics of participants and neighborhood residents

	**Participants, *N* (%)**	**Residents**^**a**^**, *N* (%)**
**Total (min, max)**	140 (10–31)	9445
**Gender**		
Men	36 (25.7%)	4745 (50.2%)
Women	104 (74.3%)	4700 (49.8%)
**Migratory background**^b^		
Dutch/Western	21 (15.0%)	4040 (42.8%)
Non-Western	119 (85.0%)	5405 (57.2%)
**Age**^c^		
18–24 years	13 (9.3%)	1395 (17.0%)
25–44 years	46 (32.9%)	3055 (37.2%)
45–64 years	58 (41.4%)	2354 (28.7%)
65 years and older	23 (16.4%)	1400 (17.1%)
Median (IQR)	49 (37–60)	NA
Mean (min, max)	48 (18–81)	NA
**Level of education**		
None	21 (15.0%)	NA
Low	44 (31.4%)	NA
Middle	32 (22.9%)	NA
High	14 (10.0%)	NA
Others/unknown	29 (20.7%)	NA
**Sexual contact**		
Heterosexual	100 (98.0%)	NA
MSM	2 (2.0%)	NA
No answer/missing	38	NA

Numbers and percentages unless stated otherwise

*IQR*, interquartile range; *max*, maximum; *min*, minimum; *MSM*, men who have sex with men; *NA*, not available

^a^Based on all residents in the selected intervention area (2019), no age selection was possible

^b^Based on participants’ and partners’ country of birth

^c^Due to the standard classification of age groups used by Statistics Netherlands, no age selection was possible for residents, and only the 5 years age groups were available. *N* (%) in the resident column reflects the age group 15 to 24 year

#### HIV testing history and perceived risk

**Fig. 1:**
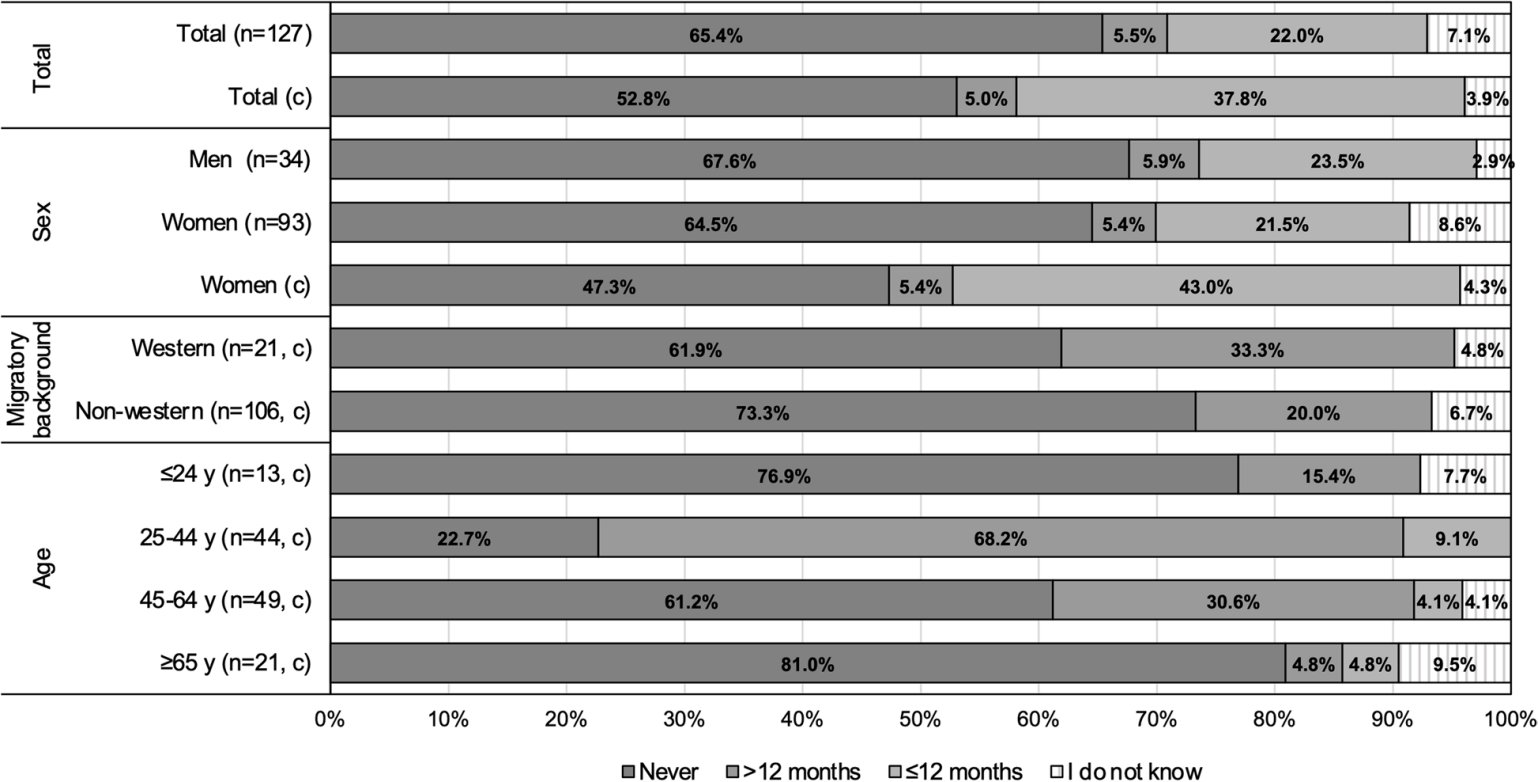
HIV testing history, by sex, migratory background, and age group (*N* = 127). Based on all participants tested for HIV during the test events that had information on HIV testing history. Rows with (c) are corrected for national HIV screening among pregnant women (pregnancy after 2003). *Abbreviations*: y, year

Figure 1 shows the HIV testing history by subgroup, and Table 2 shows whether the predefined goals regarding first-time tested and recent tested are achieved. After correction for pregnancy screening, 52.8% (*n* = 67/127) of the participants were not previously tested. The proportion never tested was highest among people with a non-Western migratory background, people aged ≤ 24 years, and people ≥ 65 years (Fig. 1). Among those tested before, this was > 12 months ago for 87.3% (*n* = 48/55). The most recent HIV test was performed mostly at the GP (29.4%), where the most frequently cited reason was medical complaints (42.2%). Nearly one-quarter (23.5%) of the participants had their most recent test at the obstetrician in relation to pregnancy.

In general, participants perceived themselves as not at risk for HIV: 57.4% (*n* = 66/115) indicated no HIV risk and 33.0% (*n* = 38/115) a very small risk. The rest (9.6%, *n* = 11/115) indicated that they did not know their risk. Future HIV risk was perceived comparably: 53.4% (*n* = 62/116) indicated no risk and 31.9% (*n* = 37/116) a very small risk. Slightly more than one in ten (*n* = 17/116) indicated that they did not know their HIV risk in the future (*You never know what will happen in the future*).

#### Health results

Table 4 shows the number of participants per health test and the proportion with an abnormal result. Overall, approximately one-third had at least one result that required a referral to their GP, of which many were not previously aware of the health problem. Specifically, more than half of the participants were unaware of their abnormal BP, while two-fifths were unaware of their abnormal BG levels, and one-third were unaware of their abnormal BMI.

**Table 4:** Number of participants per health test and the proportion with an abnormal test result

	**Participants, n/*N* (%)**	**Abnormal test result**^**a**^**, n/*N*** (%)	**Unaware of abnormal test results in advance, n/*N*** (%)
Body mass index	135/140 (96.4%)	55/135 (40.7%)	19/55 (34.6%)
Blood pressure	138 /140 (98.6%)	45/138 (32.6%)	24/45 (53.3%)
Blood glucose	137/140 (97.9%)	17/133^b^ (12.8%)	7/17 (41.2%)
HIV	134/140 (95.7%)	1/134 (0.75%)	1/1 (100.0%)

^a^Fell outside recommended ranges in national guidelines. The denominator varies per health test due to participants’ time constraints and language barrier

^b^Test result not registered for 4 tested individuals

The non-HIV tests were offered merely to improve the acceptability and uptake of HIV testing; hence, these results are not reported in detail here. The one HIV-positive case was a heterosexual woman with a non-Western migratory background. As a result, our pilot had a HIV positivity of 0.75% (95% confidence interval: 0.02–4.09%), by which the predefined goal of positivity is achieved.

#### Non-participants

Thirty-eight persons (16 men and 22 women) did not participate after receiving information about the test event. Almost 40% (*n* = 15/38) reported a practical reason for not participating (i.e., other appointments, work/internship). Around 20% (*n* = 9/38) recently visited a doctor, but not specially for HIV. The rest mentioned various reasons for non-response (e.g., scared of blood, taped hands due to boxing lessons, “not interested”).

### Effectiveness

#### Participation and reason for HIV testing

Out of the 140 participants of the test events, 134 (95.7%) opted for an HIV test. The remaining participants who did not receive an HIV test cited time constraints or there was a language barrier that prevented the staff from adequately explaining the information about HIV testing. Almost 90% (*n* = 111/127) of the participants had not undergone an HIV test either in the past 12 months (*n* = 28/111) or ever before (*n* = 83/111).

When participants were asked for their reason to test for HIV during the test event, 88.6% (*n* = 124/140) gave at least one reason. Most frequently mentioned reasons were to be certain (47.6%), no special reason (24.2%), because it is free-of-charge (16.9%), and because multiple tests were offered (15.3%). Participants also indicated the convenience of the test event: *At the GP you only test if you are ill, here you just enter, and you can test. This is nice and easier*.

#### Acceptability of HIV testing

All participants expressed willingness to undergo HIV testing. Moreover, participants reported a higher likelihood of undergoing an HIV tests when initiated by their GP (72.2%, *n* = 84/118), as opposed to them having to request it themselves (38.1%, *n* = 45/118). The majority of participants (73.8%) indicated that they would prefer to receive a reason from their GP for recommending the HIV test, but they would still undergo the test regardless (*He doesn’t just ask*, *He probably has a good reason*). More than half (54.7%) of the participants suggested that HIV testing should always be free-of-charge, like at the test events.

#### Stigma reduction and increasing knowledge

The staff and community leaders observed that participants engaged in open conversations about HIV and health in general. They suggested that such discussions may contribute to reducing the stigma and taboo associated with HIV. The staff and community leaders reported that test events contributed to increased knowledge and openness, and helped reduce HIV-related stigma by normalization and taboo reduction. This was facilitated by the game provided during waiting time. One community leader said: *A lot of people did not know what HIV was, but because of today they know*. This was further supported by the participants themselves. One participant reported: *I didn’t know about HIV, but now I do*, while another noted that: *When people talk more about it [HIV], taboo decreases. This [test event] also helps!* Additionally, both the staff and community leaders mentioned that the presence of a CBHT in general positively contributed to the attitudes and knowledge about HIV/PLHIV.

### Adoption

Establishing and maintaining contact with community organizations required frequent communication, persistence, and the importance of finding the right person. The most effective approach to establishing contact with the organizations was through in-person visits, rather than e-mail or telephone communication. This informal and personal interaction also facilitated the development of a network and trust by the organization.

The involvement of the community leaders and professionals of local organizations was essential for the successful adoption of the test events. Community leaders and professionals from local organizations gave advice about the design and implementation of the test events. This process created local support for the test events. A wide range of local organizations were interviewed, e.g., healthcare organizations, various types of community support organizations, organizations facilitating social gatherings or courses, and sports facilities. Although some interviewed organizations were surprised about the topic of HIV, they were all willing to cooperate by opening up their location for the test events and/or by promoting the events through hanging posters, distributing flyers, and via their social media platforms. Test event locations were chosen based on features, such as having separate rooms or the possibility to place the test bus in front of the location, as well as the population visiting these organizations (e.g., diverse group, young people). The initial plan was to involve local health professionals as well, such as general partitioners or district nurses. However, we did not succeed to involve them in the test events, because of their lack of time and commitment to other priorities.

The facilities at the test events at the community center and support organization were rated as sufficient by the staff. The boxing school was found to be inadequate in terms of providing sufficient privacy and workspace that met professional standards, as the pre-arranged spaces were occupied by athletes. In addition, young people did not participate as they only stayed for their training. Staff questionnaires and interviews with community leaders were among others used to evaluate which people were reached during the pilot. This guided the planning of future test events, including reaching out to involved and new organizations serving different population groups than those already reached. Test events to reach other population groups, including men and youngsters, via a barbershop and a youth organization, were canceled due to the COVID-19 pandemic.

### Implementation

#### Planning and execution

The test events were extensively planned. Whenever possible, the date and time of the test events were scheduled to coincide with other events taking place at the location, ensuring that the target group would be present. The test events were generally carried out as planned. The high level of flexibility of the staff ensured that any problems that arose on the day of the test event were resolved swiftly (e.g., last-minute changes to rooms due to them being occupied by other events). The significance of having multiple test events at a single community organization was recognized as it promoted better collaboration between the organization and staff and facilitated the planning of future test events. Moreover, the test bus helped in raising awareness among local residents about the test events. Additionally, the staff indicated that maintaining a consistent team composition throughout the test events was advantageous for fostering interaction, cooperation, and contact with the community (leaders) and residents.

One of the major challenges was the labor-intensive nature of the approach, particularly during the preparation phase. This involved searching for benevolent organizations and individuals for the needs assessments, conducting the needs assessment, maintaining communication with stakeholders, developing materials (e.g., questionnaires, posters, and leaflets), and checking suitable locations. Another challenge was managing waiting times during the test events. Waiting time was the lowest-scored item by participants, with 3.5% (*n* = 4/116) rating it as not good and 13% (*n* = 15/116) as neutral. The staff was also critical about the waiting time when a high volume of concurrent walk-ins resulted in longer than desired waiting times. Language barrier was the main challenge mentioned by the staff, particularly during the questionnaire administration and, to a lesser extent, during other stops. The staff expressed concerns that the lack of understanding could have negatively affected the comprehension of the procedure and the reliability of the participants’ answers. In cases where the staff felt the participants’ understanding was low, (hypothetical) questions were sometimes skipped. In some instances, participants translated for each other, which staff felt compromised privacy. However, in general, participants did not express concerns about language barriers and privacy, even when the staff attempted to intervene to improve privacy (for example, when a surveyed participant expressed a desire for other participants to stay).

Recruitment was also a challenge. Both the staff and community leaders were dissatisfied with the overrepresentation of Moroccan and Turkish women above 40 years. One community leader explained: *Men did not want to participate because the location was predominately occupied with women and this scared the men*. However, a relatively homogenous population was also considered as beneficial, as participants had in-depth discussions on health and HIV with each other and the staff.

#### Experiences and feedback

Over 80% of the participants rated the various aspects of the pilot as (very) good. Furthermore, they were appreciative of the provided actions in their community: *I am happy. This event came here out of nowhere! And happy with the test results!* Community leaders also expressed high satisfaction with the program and the attention to “their people.” Both the staff and community leaders emphasized that the pilot’s core elements (within the community, general health check, and free-of-charge) helped to reach first-time testers and did not encounter stigma-related issues to HIV testing. In addition, community leaders and participants were positive about the game and indicated they learned new information. The staff indicated that participants who played the game before the first stop at the test event were better informed about HIV.

The involvement of community leaders was considered valuable, especially in recruiting participants. Although it was indicated as labor-intensive by the staff, continuous contact with the community leaders, and repeated presence at locations, created a network and trust, which benefited the execution of the test events. Community leaders appreciated the contact and involvement, especially during the input phase. None of the community leaders found that the preparation and execution of the test events (time) demanding. Community leaders did feel responsible for recruitment.

### Maintenance

All community organizations that provided their location for the test events were willing to facilitate in future test events. Participants also indicated that they wanted to participate again in the future (*continue with this initiative*). During the test events, some participants called family and friends and urged them to come and test. Several participants wrote that there should be more test events, with one participant explaining: *people go to the doctor too late*. Despite the success and perceived usefulness of the test events for the residents, the staff expressed hesitancy to continue due to the labor-intensive, and therefore costly, nature of the events. Nonetheless, the staff acknowledged the value of a wide range of health tests, including HIV testing. A collaboration with other health programs and/or improve HIV testing at GP was seen as more sustainable.

## Discussion

This study showed the pilot CBHT intervention, a combination of an HIV test with other health checks, to be feasible and acceptable. While not all predefined goals were achieved, the pilot was well-rated by all parties involved and successfully reached many first-time and not recently tested individuals with low perceived HIV risk. We found one positive HIV participant (HIV positivity of 0.75%).

The developed CBHT intervention met most recommendations of the stakeholders and was considered as low-threshold due to its features such as decentralization, anonymity, and free-of-charge rapid HIV testing. This is supported by other studies [25–29]. Participants did not report any doubts about HIV testing, which seems to underpin the low-threshold setting. Non-participants mainly cited practical reasons for not participating (e.g., other appointments, work). Another stakeholder recommendation was to increase HIV knowledge. Although not quantitatively measured, the educational game was well received and may have positively impacted knowledge and attitudes about HIV/PLWH. Both increasing knowledge about HIV and combining HIV testing with other non-stigmatized health tests can help to normalize and reduce the stigma related to HIV and HIV testing [9, 30–37]. No instances of HIV-related stigma were observed by the staff and the community leaders during the test events.

CBHT can increase the likelihood of reaching and testing key populations, particularly, in areas where they are concentrated. This suggests that more geographically targeted approaches may be effective in improving HIV testing uptake [3, 4, 29]. In The Netherlands, people with a non-Western migratory background are an important key population for HIV, alongside men who have sex with men (MSM) [3, 18]. To target this population, we selected an area with a relatively high HIV prevalence and a relatively large proportion of people with a non-Western migratory background. Although the participants’ sociodemographics did not reflect the neighborhood’s demographics, we were able to reach an even larger proportion of non-Western participants, one of which tested HIV positive. This woman, like most of the participants, perceived low risk for HIV and would probably not have tested until symptoms appeared, potentially leading to delayed diagnosis and further spread of HIV. Compared to MSM, people with a non-Western background in The Netherlands have a higher proportion of late-stage HIV infections and undiagnosed HIV [3], making this population especially important in finding the last cases in this phase of the HIV epidemic. The high proportion of late-stage HIV infections among non-Western people also may indicate that they may not be adequately reached by regular healthcare services such as the GP and SHC, the two main providers of HIV tests in The Netherlands. CBHT approaches like ours can effectively reach individuals with low-estimated risk and first-time testers, especially those who are not likely to utilize other healthcare services [6, 8]. We were able to reach a significant proportion of first-time testers (52.3%) and not recently tested for HIV (87.3%). However, CBHT is usually not conducted on a frequent, regular, and widespread basis and is costly. Therefore, improving proactive HIV testing at regular healthcare services seems more practical and sustainable [28]. This was also indicated by the staff in our study.

Our study observed HIV test opportunities at the GP; 71.2% of the participants would be willing to test for HIV if their GP offered the test, compared to only 38.1% who would request a test themselves. Although most participants indicated a preference for a reason for the HIV test recommendation from their GP, we indirectly found that people accept an HIV test if it is offered as none of the participants declined the HIV test, even without any prior advice. This highlights the importance of HIV testing being offered proactively. However, it is known that GPs currently adhere insufficiently to HIV testing guidelines, even during STI consultations with high-risk patients [38–40]. Guideline adherence will be even more difficult if a patient does not belong to known key populations or if they consider themselves not at risk. Our study identified one participant who tested positive for HIV and was not notified by her partner. This exemplifies that partner notification is another effective method for the timely detection of new HIV infections, though its implementation is currently insufficient [40, 41].

We showed that the performed pilot was generally well-received and feasible to conduct, with some important lessons learned. First, this approach is very labor intensive, and therefore costly, particularly in the preparatory phase (e.g., find benevolent organizations, investment in and stay in contact with stakeholders, need assessment, material development, prior visits to check the locations at the planned day/time). However, investing in this phase was indicated as crucial for the success of the intervention and can partly be compensated by increasing the number of test events. Second, active involvement of local community organizations and staffs’ repeated presence was found essential for tailoring interventions for community needs, for location usage, and for recruitment, but also to build trust and social cohesion. The involved organizations felt that their voices were heard and that they had a sense of shared responsibility in the recruitment and execution processes. Third, evaluation among all involved parties is valuable for gaining insight into potential in-between adjustments. Our study found that major concerns expressed by the test team (e.g., language problems and privacy), were not shared by most participants. Finally, the general health check also provides opportunities for collaboration with other health organizations in the neighborhood. Collaboration could reduce costs and provide benefits for specialized health advice. However, establishing these collaborations can be challenging, as health professionals that we approached did not want to corporate either because of lack of time or other priorities.

### Limitations

Our study had several limitations. First, the results may not be generalizable as this is a pilot project with a small sample size from a specific geographical area. In addition, the composition of the participants is affected by the organizations where we performed the test events and the day and time of the events. While we used different locations, days, and parts of the day, we predominantly reached middle-aged women. The staff suggested other solutions, such as connecting test events to other activities (e.g., sports) and using a more diverse group of community recruiters. Attempts to reach more men and youngsters via a barbershop and a youth organization, both of which had expressed willingness to participate, were canceled due to the COVID-19 pandemic. Second, we did not collect detailed HIV risk factor information from participants, making it difficult to assess the underlying HIV risk. Our aim was to offer testing at the community risk level as opposed to the individual risk level. Moreover, adding questions about HIV risk would have increased the barrier to participate; the staff was concerned that it could jeopardize privacy and further increase the already time-consuming questionnaire. Third, systematic collection of non-response was not always possible due to the multiple tasks of the test team members and the walk-in setting. Finally, the questionnaire was orally administrated in Dutch or with an improvised translation into English. If there was still a language barrier, simplified additional explanation was given or participants translated for each other. This may have affected the reliability of the questionnaire answers. Additionally, not all questions were answered by all participants. The staff members proposed different solutions to address language barriers, such as multilingual staff or a telephone interpreter. However, not all staff preferred these options, as there was a diverse range of languages spoken by the participants, and using a telephone interpreter was conceived as unfeasible.

## Conclusions

Offering decentralized anonymous free-of-charge rapid HIV testing in combination with other more general health tests was feasible, accepted, and effective to test not (recently) tested persons. The approach appeared to positively impact attitudes and knowledge about HIV/PLHIV according to the staff and community leaders. However, there were some concerns about the labor-intensive nature of this approach, and whether it is worth the investment to find the last unidentified HIV cases. As we observed multiple health problems among the participants, collaborations with other health programs and professionals could help to reduce costs, share expertise, and further normalize and destigmatize HIV (testing). However, in the phase of micro-elimination of HIV, CBHT may be a suitable supplement to more sustainable and cost-effective methods, e.g., proactive HIV testing by GPs and partner notification.

## Data Availability

All relevant questionnaire data are within the manuscript. Due to the detailed and sensitive personal information and the relatively small geographical area the study has been conducted, we are not able to make our data publicly available to keep the privacy of the study participants. To achieve access to the data used in this paper for research purposes, please contact the corresponding author. Requested data will be de-identified before sharing.

## Abbreviations

AIDS: Acquired immunodeficiency syndrome
BG: Blood glucose
BMI: Body mass index
BP: Blood pressure
CBHT: Community-based HIV testing
FGD: Focus group discussion
GP: General practitioner
HIV: Human immunodeficiency virus
PHS: Public Health Service
MSM: Men who have sex with men
PLHIV: People living with HIV
